# Book Reviews

**Published:** 1876-10

**Authors:** 


### *BOOK REVIEWS.*

"LIVER COMPLAINT, NERVOUS DYSPEPSIA AND HEADACHE; Their Causes, Prevention, and Cure." By M. L. Holbrook, M. D. This is a neat volume of 140 pages, and in it the author describes the functions and disorders of the liver in their relation to many forms of indigestion. The concluding portion of the book is devoted to a consideration of the causes and relief of headache. The language of the book is simple, as it should be in a work designed for thoughtful hygienists rather than for professional readers. Its perusal could hardly fail to benefit the class of sufferers for whom it is mainly intended.

Dr. Holbrook is a prominent hygienist, and has written largely on food and other topics of great interest to mankind. He is, we are glad to know, not a "Grahamite" in such a sense as to commend the branny trash which Sylvester Graham strove to force down the throats of his misguided followers. He knows—as all who have studied the chemistry of foods must know—that our flour is robbed of the most of its nourishment in the process of manufacture; but he does not on this account ask his readers to swallow all manner of husks and hulls with the better portion of the cereal grains. We were glad to notice in the last issue of the *Herald of Health*, edited by this gentleman, a cordial and hearty indorsement of the better food-philosophy of which the HEALTH FOOD COMPANY is the leading exponent. This new philosophy excludes insoluble substances from cereal foods, and declares bran to be pernicious and dangerous.

This book on the liver and its disorders will be of great value to all who would gain a more intimate knowledge of "the house we live in," and the proper methods for its repair by hygienic means.

